# Supplementary material for: Splice-Junction-Based Mapping of Alternative Isoforms in the Human Proteome
Source: Cell Rep. Author manuscript; Available in PMC 2020 Jan 15. (PMC6961840; doi:10.1016/j.celrep.2019.11.026)
Supplement: 2 [file NIHMS1546469-supplement-2.zip › DF1/PXD010154/AdrenalGland/AdrenalGland_3_CDV3_VQAMQISEKEEDDNEKR.pdf]

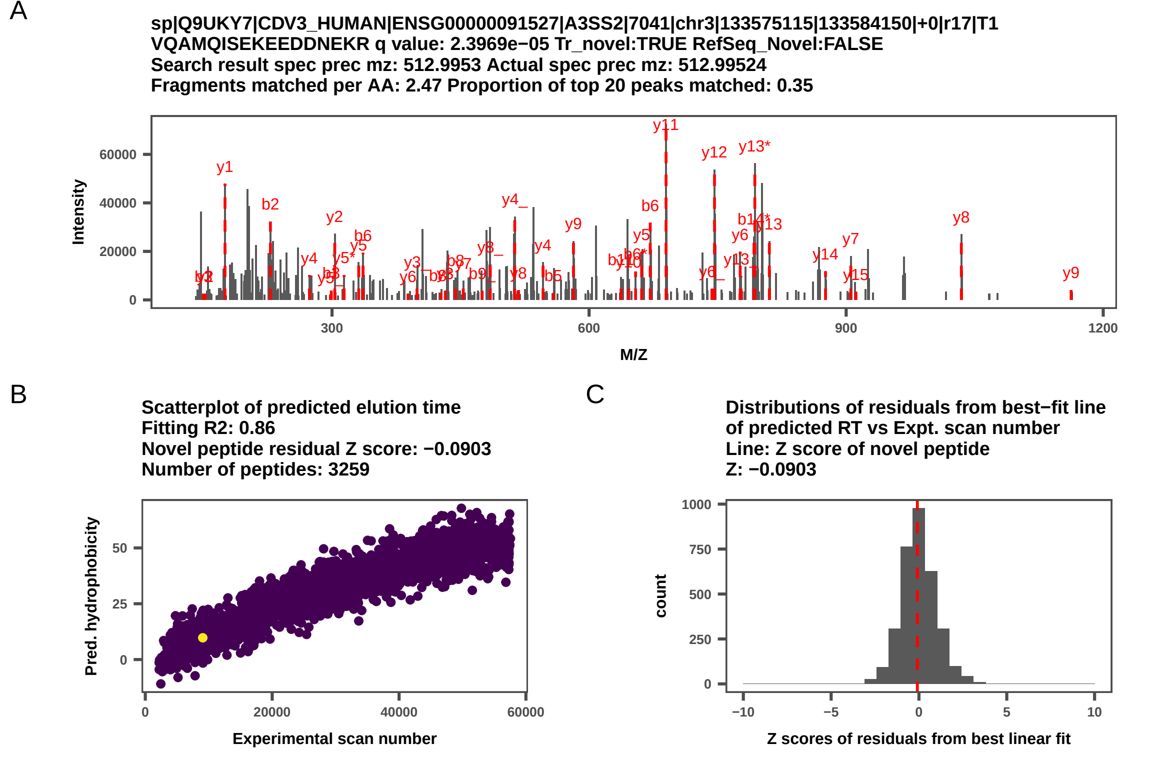
A
sp|Q9UKY7|CDV3_HUMAN|ENSG00000091527|A3SS2|7041|chr3|133575115|133584150|+0|r17|T1
VQAMQISEKEEDDNEKR q value: 2.3969e−05 Tr_novel:TRUE RefSeq_Novel:FALSE
Search result spec prec mz: 512.9953 Actual spec prec mz: 512.99524
Fragments matched per AA: 2.47 Proportion of top 20 peaks matched: 0.35
Intensity
60000
40000
20000
0
y1
b2
y2
b6
y5
y4
y4_
y9
b6
y11
y12
y13*
y8
y7
y14
y15
y9
300
600
900
1200
M/Z
B
Scatterplot of predicted elution time
Fitting R2: 0.86
Novel peptide residual Z score: −0.0903
Number of peptides: 3259
Pred. hydrophobicity
50
25
0
0
20000
40000
60000
Experimental scan number
C
Distributions of residuals from best−fit line
of predicted RT vs Expt. scan number
Line: Z score of novel peptide
Z: −0.0903
count
1000
750
500
250
0
−10
−5
0
5
10
Z scores of residuals from best linear fit
